# The Application of Cold-Induced Liquid–Liquid Extraction for the Determination of 4-Methylimidazole in Tea and Associated Risk Assessment for Chinese Tea Consumers

**DOI:** 10.3390/toxics11110916

**Published:** 2023-11-09

**Authors:** Shaohua Li, Lian Wang, Dawei Chen, Hong Li

**Affiliations:** 1 Tea Science Research Institute, Wuyi University, Wuyishan 354300, China; lsh@wuyiu.edu.cn; 2 Chengdu Centre for Disease Control and Prevention, Chengdu 610044, China; septwolvesnjwl@163.com; 3 School of Public Health, Jinzhou Medical University, Jinzhou 121001, China; 4 NHC Key Laboratory of Food Safety Risk Assessment, Chinese Academy of Medical Science Research Unit (No. 2019RU014), China National Center for Food Safety Risk Assessment, Beijing 100021, China

**Keywords:** tea, 4-methylimidazole, analytical method, exposure assessment, sample preparation, health risk

## Abstract

4-Methylimidazole (4-MEI), as a Maillard reaction product, often occurs in heat-processed food. Due to its widespread occurrence and strong carcinogenicity in food and beverages, 4-MEI has received attention from regulatory organizations and consumers. Some studies have reported the occurrence and exposure of 4-MEI in food, but few studies have involved traditional tea beverages, which is related to the limited analytical methods currently being influenced by complex tea matrices. For this issue, this study presents a simple, reliable, and highly sensitive analytical method for the determination of 4-MEI in tea using liquid chromatography–high resolution mass spectrometry. By means of this method, a total of 570 tea samples from typical tea-producing regions in China were monitored for contamination of 4-MEI. The results showed that the average 4-MEI level (136 μg/kg) in oolong tea was significantly higher than that in other types of tea samples. Based on contamination levels and tea consumption data in China, the daily intake doses (0.04–1.16 μg/day) of 4-MEI among tea consumers were obtained. As a result, the health risk of Chinese tea consumers consuming 4-MEI alone through tea consumption is relatively low, but the overall intake level of 4-MEI in other foods cannot be ignored.

## 1. Introduction

Tea is the second most popular non-alcoholic beverage in the world, second only to water [1,2], and has potential health benefits [3]. Tea can be divided into green tea, dark tea, oolong tea, black tea, white tea, and yellow tea based on different production processes [4]. Although tea has antioxidant, antidiabetic, and neuroprotective effects [5,6,7], it also contains certain hazardous compounds that may pose a threat to consumer health. Hazardous compounds, such as pesticides [8], polycyclic aromatic hydrocarbons [9], heavy metals [10], and rare earth elements [11], have been found in tea.

The Maillard reaction plays an important role during the thermal processing of tea (Figure S1). The thermal processing of tea is the cause of the Maillard reaction, in which reducing sugars or aldehyde groups from tea react with amino acids to form heterocyclic compounds, including pyrazines, furans, pyrroles, and other products, which are responsible for producing the desired aroma and color of the tea [12]. The Maillard reaction can also produce additional by-products, such as 4-methylimidazole (4-MEI). 4-MEI is a low molecular weight compound containing nitrogen heterocycles, mainly present in caramel-colored foods (Classes III and IV) [13,14,15,16,17,18], such as soft drinks, cola drinks, black beer, vinegar, soybeans, etc. 4-MEI can also be found in naturally processed foods without a caramel color [19,20,21], such as tea, coffee, and grains. It is generally considered to be formed by the Maillard reaction [5,22]. 4-MEI has been confirmed as a 2B carcinogen substance by the International Agency for Research on Cancer (IARC, 2011) [23] and the National Toxicology Program (NTP) [24]. In addition, the Joint Expert Committee on Food Additives (JECFA) of the FAO/WHO has also limited the use of food additives containing 4-MEI [25]. The European Union (EU) and the United States (US) have set the maximum level of 4-MEI in Class III [26] and Class IV [26,27] caramel-colored foods to 200–250 mg/kg, and the “no significant risk level” in California (OEHHA) is less than 29 µg/day [28]. However, limited data on the occurrence of 4-MEI in tea, exposure assessment, and health risks for tea consumers are present, which are necessary for developing and revising maximum allowable levels of daily human intake to protect the health of drinkers.

Several methods for the analysis of 4-MEI in foodstuffs have been reported, mainly using gas chromatography–mass spectrometry (GC-MS) [29] and liquid chromatography–mass spectrometry (LC-MS) [18,23,30,31,32,33]. However, the high amounts of polyphenols, alkaloids, amino acids, and pigments in the tea matrix seriously interfere with the analysis of 4-MEI in tea by LC-MS. Therefore, sample preparation to remove caffeine, polyphenols, and preconcentrations of 4-MEI is the key step. Generally, solid-phase extraction (SPE) [30], supercritical fluid extraction (SFE) [31], dispersive liquid–liquid micro-extraction (DLLME) [32], and dispersive micro-solid-phase extraction (DMSPE) [33] techniques have been used for the extraction and cleanup of 4-MEI. However, the SPE technique consumes large volumes of solvents with its tedious procedures; SFE requires a specialized instrument, while DLLME has mainly been applied to aqueous samples. DMSPE [33], as a modified QuEChERS method, is not able to effectively remove complex interfering substances from tea. Recently, cold-induced liquid–liquid extraction (CI-LLE) [34,35], as a modified LLE method, was conducted by distributing a homogeneous acetonitrile/aqueous extract into two phases at freezing temperature without salts. This CI-LLE method has been applied to protein purification [36] and the extraction of pesticides, veterinary drugs, and perchlorate [37]. To our knowledge, this technique was first applied to the extraction of 4-MEI and the cleanup of tea matrices.

This study aims to: (a) develop a new analytical method for the determination of 4-MEI in tea based on the CI-LLE technique; (b) investigate the concentrations of 4-MEI in all types of tea and the transfer rate from dry tea into tea infusion; and (c) estimate the tea intake exposure to 4-MEI of tea consumers and assess health risks of 4-MEI to drinkers.

## 2. Materials and Methods

### 2.1. Chemicals

The standard 4-MEI was supplied by AccuStandard, Inc. (New Haven, CT, USA), and the isotope-labeling standard 4-methylimidazole-d6 (4-MEI-d6, IS) was purchased from C/D/N Isotopes (Pointe-Claire, Quebec, QC, Canada). LCMS-grade acetonitrile (MeCN) was obtained from Fisher Scientific (Fair Lawn, NJ, USA) and LC-grade ammonium acetate was purchased from Tedia (Weston, OH, USA). Deionized water (H_2_O, Milli-Q) was used throughout this study.

### 2.2. Tea Sampling and Preparation

Tea (*Camellia sinensis*) samples (*n* = 570) were obtained from typical tea-producing regions in China, including green tea (*n* = 109, GT), oolong tea (*n* = 237, OT), black tea (*n* = 80, BT), white tea (*n* = 56, WT), dark tea (*n* = 27, DT), yellow tea (*n* = 10, YT), and scented tea (*n* = 51, ST). Furthermore, a total of 77 oolong tea samples from the same region (Wuyishan, Fujian) were divided into two production processing methods, including low-temperature baking treatment (*n* = 34) and high-temperature baking treatment (*n* = 40) to evaluate the temperature impact on the formation of 4-MEI during tea processing. Briefly, low-temperature baking treatment involved roasting at 105–110 °C for 6 h, and high-temperature baking treatment involved roasting at 140–145 °C for 13 h. All of the tea samples were crushed by a high-speed crusher after freezing with liquid nitrogen, passed through an 80-mesh sieve, and stored at room temperature.

The homogenized tea samples (1.0 g) were measured, and 20 μL of 4-MEI-d6 (1 mg/L) was spiked and vortexed for 30 s by a vortexer (vortex Genie 2, Scientific Industries, NY, USA) and then placed for 30 min. A 10 mL MeCN-H_2_O (3:1, *v*/*v*) mixture was used to make the samples, which were extracted ultrasonically for 30 min (Kunshan Shumei KQ-100DB ultrasonic cleaning machine, 40 KHz; 100 W; 40 °C, Kunshan Ultrasonic Instrument Co., Ltd., Kunshan, China). After centrifuging the extract at 9000 rpm for 3 min at room temperature (SIGMA 2-16KL centrifuge, Sigma, Darmstadt, Germany), the supernatant was transferred to a 15 mL Eppendorf tube and was frozen for 60 min in a −20 °C freezer. After the cold-induced phase separation, the lower H_2_O layer was diluted five-fold with MeCN and then filtered through a syringe filter for LC-HRMS analysis.

### 2.3. Instruments

The determination of 4-MEI was described in our previous work [33]. Briefly, UHPLC (Dionex, Ultimate 3000 RSLC system, Sunnyvale, CA, USA) separation was performed using a BEH HILIC column (100 × 2.1 mm, 1.7 μm, Waters, MA, USA) with the column temperature set at 40 °C. The mobile phase was composed of MeCN and 5 mM ammonium acetate in H_2_O with isocratic elution (90:10, *v*/*v*) at 0.4 mL/min. HRMS (Thermo Q Exactive, Bermen, Germany) equipped with an electrospray ionization source (ESI) was set in positive ESI mode with a spray voltage of 3800 V and ion source temperature of 320 °C. N_2_ was used as sheath gas and auxiliary gas set at 40 and 10 arbitrary units (arb), respectively. Parallel reaction monitoring (PRM) mode was operated at a resolution of 70,000 full wave at half maximum (FWHM). The other relevant parameters can be found in our previous work [33].

### 2.4. Optimization of Sample Preparation

One-step enrichment and cleanup of 4-MEI was completed in this study by using cold-induced liquid–liquid extraction (CI-LLE) for the MeCN/H_2_O extract when the temperature was set at −20 °C. The optimum conditions, including the proportion of the MeCN/H_2_O mixture, the freezing time, and temperature, were optimized by evaluating the enrichment factor and extraction efficiency of 4-MEI. The enrichment factor was calculated from the concentration ratio of 4-MEI found in the lower aqueous phase and that in the sample extract before phase separation, while the extraction efficiency was determined using the amount of 4-MEI between the lower aqueous phase and the sample extract. Herein, partition experiments of the cold-induced phase separation were performed in 5 mL Eppendorf tubes with 5 mL of 4-MEI standard solution (10 µg/L) in different proportions of MeCN/H_2_O mixture. In this way, the optimum proportion of MeCN/H_2_O mixture was selected based on overall consideration of the enrichment factor and extraction efficiency of 4-MEI. Additionally, the effect of the freezing time in the CI-LLE procedure on the complete partition of 4-MEI in two phases was also discussed as the time varied from 30 min to 90 min.

### 2.5. Method Validation

Method specificity was conducted by preparing and analyzing blank tea samples to ensure that the determination of 4-MEI and 4-MEI-d6 was not disturbed by matrix interferences. Linearity was validated by plotting the calibration curves, with the 4-MEI concentration as the x-coordinate, and the 4-MEI/4-MEI-d6 peak area ratio as the y-coordinate. The concentrations of ten calibration points ranged from 0.1 µg/L to 100 µg/L, with each concentration containing 4-MEI-d6 at 2 µg/L. Due to the obvious background noise in blank tea samples for 4-MEI analysis by HRMS, the limit of detection (LOD) and limit of quantitation (LOQ) were calculated as 3-times and 10-times signal-to-noise ratios (S/N), respectively. Accuracy and precision were validated by spiking three level amounts of standards into the blank tea sample, each level with six replicates, and calculating the extraction recovery (%) and relative standard deviation (RSD%). A blank tea sample was obtained during testing, which cannot contain 4-MEI, requiring it to be lower than the LOD of this study. Considering the complex of tea matrices, matrix effects (MEs) still needed to be evaluated even if an available isotope-labeling standard was used in this study. The ME evaluation was conducted according to our previously reported method by comparison of the slope ratio between matrix-blank matched and matrix-free calibration curves without 4-MEI-d6 calibration [33]. When the slope ratio is between 0.8 and 1.2, this means that the method has a weak matrix effect, which can be neglected. On the contrary, it has a certain matrix enhancement or suppression effect.

### 2.6. Quality Assurance and Statistic Analysis

A low-concentration spiked sample was analyzed in each batch analysis (20 samples) to ensure the reliability of the results throughout the analytical sequence. Additionally, a solvent blank was analyzed to track potential carryover or systemic contamination prior to the calibration curve, after the calibration curve, or after each batch analysis. As expected, the recoveries of spiked samples in each batch analysis needed to fall between 70% and 120%, and the solvent blank fell below the LOD for 4-MEI. All of the samples were analyzed in duplicate, and the relative average deviation should be less than 20%.

All of the data were processed and calculated with either Microsoft Office Excel 2007 or GraphPad Prism v. 7.04. Differences between groups were determined through one-way ANOVA, with post-hoc analysis conducted using Dunn’s test for multiple comparisons. Non-parametric Mann–Whitney U tests were carried out to compare the 4-MEI levels for different processing temperatures via two-group comparison. A two-sided *p*-value of <0.05 was considered statistically significant.

### 2.7. Transfer Rate of 4-MEI during Tea Brewing

In this study, tea-positive samples of two different concentration levels (76.2 µg/kg and 687 µg/kg) were selected to evaluate the transfer rate of 4-MEI from dry tea to its infusion. Tea brewing was performed by the sensory evaluation method in China. Briefly, 20 mL of boiling H_2_O was poured into 1.0 g of the tea sample. After a certain brewing time (5 s, 2 min, 3 min, and 5 min), the tea infusion was filtered and cooled down to yield the tea infusion sample from the first to the fourth infusion. Finally, the brewed tea was dried after 5 min, and the 4-MEI was re-extracted to yield the final tea extract. The transfer rate (%) of 4-MEI from the tea into the infusion was calculated by comparison of the amount ratio.

### 2.8. Consumption Data and Dietary Intake Calculations

The daily intake doses of 4-MEI by Chinese tea consumers were evaluated by the semi-probabilistic approach as described by Fierens et al. [38]. In brief, this approach used distributions of daily consumption data in combination with specific fixed concentrations of 4-MEI in tea samples. The tea consumption data were obtained from Cao et al.’s study based on the China National Nutrient and Health Survey conducted in 2002 [39]. The mean, 50th (P50), 90th (P90), 95th (P95), and 97.5th percentiles (P97.5), and maximum (P100) of the daily consumption data of tea were used in this study. As for the specific fixed concentrations of 4-MEI, three different scenarios were used based on the used 4-MEI concentration data, such as mean, median, and maximum values. Daily intake dose was estimated based on multiplying specific fixed concentrations of 4-MEI in tea by the consumption data: DID = C_i_ × T_r_ × M/1000, where DID is the daily intake dose (µg/day), C_i_ is the concentration of 4-MEI in dry tea (ng/g), T_r_ is the transfer rate of 4-MEI from tea to infusion (%), and M is the daily consumed amount of dry tea (g/day).

## 3. Results and Discussion

### 3.1. Optimization of Sample Preparation

**Figure 1:**
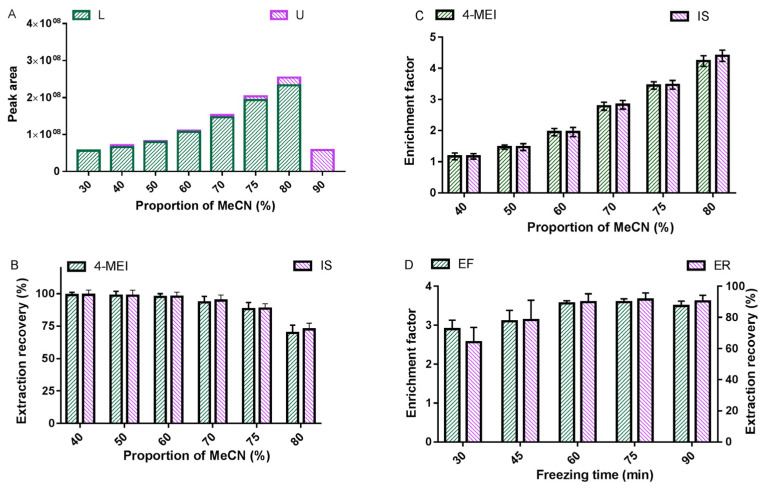
Optimization of sample preparation: (**A**) distribution of 4-MEI in the upper (U) and lower (L) phases under different acetonitrile–water ratios; (**B**) enrichment factor of 4-MEI and 4-MEI-d6 (IS) under different acetonitrile–water ratios; (**C**) extraction recovery of 4-MEI and 4-MEI-d6 (IS) under different acetonitrile–water ratios; and (**D**) enrichment factor (EF) and extraction recovery (ER) of 4-MEI under different freezing times.

In our previous studies, we found that the CI-LLE procedure is helpful for one-step purification and enrichment of organic contaminants in complex matrix samples, such as perchlorate and contaminants with different polarity [34,37]. The principle is that the target analyte is enriched in the upper organic phase by phase separation induced by low-temperature freezing. To understand the distribution coefficient of 4-MEI between the upper MeCN and lower aqueous phases, the CI-LLE procedure was used to evaluate the distribution of 4-MEI in different proportions of MeCN/H_2_O solution. Figure 1A shows that 4-MEI was more easily distributed in the lower aqueous phase in whatever proportion of MeCN/H_2_O solution due to its high polar structure and strong water solubility. In addition, it can be seen in Figure 1A that the peak area of 4-MEI in the lower aqueous phase increased with the increase in the MeCN/H_2_O ratio, which is mainly due to the continuous decrease in the volume in the lower aqueous phase, resulting in the increase in the concentration of 4-MEI. However, at 30% and 90% of the MeCN/H_2_O ratio, the two phases formed in the CI-LLE procedure all converged into one phase, which could not form effective phase separation. To this end, we further investigated the absolute extraction recovery and enrichment factor of 4-MEI and its isotope internal standard 4-MEI-d6 (IS) in 40–80% of MeCN/H_2_O solution. The results showed that, firstly, no matter what proportion of MeCN/H_2_O solution was used, 4-MEI and 4-MEI-d6 had similar trends for absolute extraction recovery and enrichment factor in the CI-LLE procedure (Figure 1B,C), suggesting that 4-MEI-d6 was suitable for recovery calibration in the CI-LLE procedure. Secondly, it can be seen from Figure 1C that the enrichment factor of 4-MEI enriched in the lower aqueous phase also increased with the increasing proportion of MeCN/H_2_O solution. However, by observing the absolute extraction recovery of 4-MEI in Figure 1B, it can be seen that when the proportion of MeCN/H_2_O solution reached more than 70%, the absolute extraction recovery of 4-MEI decreased slightly, which is mainly due to the increasing volume of the upper MeCN phase, improving the distribution of 4-MEI in the upper MeCN phase. Therefore, considering the enrichment factor and absolute extraction recovery of 4-MEI in the lower aqueous phase, 75% of the MeCN/H_2_O mixture was selected as the extraction solvent of 4-MEI in the tea samples. Under this condition, the enrichment factor of 4-MEI in the lower aqueous phase can reach ~3.5 times, and the absolute extraction recovery is nearly 90%.

Previous studies found that the two-phase formation of the CI-LLE procedure is related to the freezing-induced temperature [34,37]. With the temperature from −20 °C to −80 °C or using lower temperature liquid nitrogen (−150 °C), the time of induced phase separation will be reduced from hours to seconds. In this study, considering that a −20 °C refrigerator is easy to obtain in a routine analytical laboratory, −20 °C was used as the freezing-induced temperature, and the freezing-induced time was further optimized at this temperature. Figure 1D shows that the enrichment factor and absolute extraction recovery of 4-MEI reached a stable optimal state when the freezing-induced time reached 60 min. Therefore, 75% of MeCN/H_2_O solution was used as the extraction solvent of 4-MEI, and the temperature of −20 °C was used for 60 min to induce phase separation.

### 3.2. Method Validation

The established method was validated for its specificity, linearity, LOD, LOQ, accuracy, precision, and matrix effect. For specificity evaluation, the analysis of 4-MEI and 4-MEI-d6 in a blank solvent and a blank tea sample was performed to test the interferences using the developed method. No significant interference peaks were detected near the retention time of analytical targets. Table 1 shows the validated results with superior linearity (R^2^ = 0.9994) and detection performance (0.3 µg/kg and 0.8 µg//kg for LOD and LOQ, respectively), good extraction recovery of 92.5–109% at three different spiked levels, and good precision between 4.3% and 8.6%. Additionally, a weak matrix effect (0.91 ± 0.04) was observed for the analysis of 4-MEI in tea samples using the proposed method.

### 3.3. 4-MEI Concentrations in the Tea Samples

**Figure 2:**
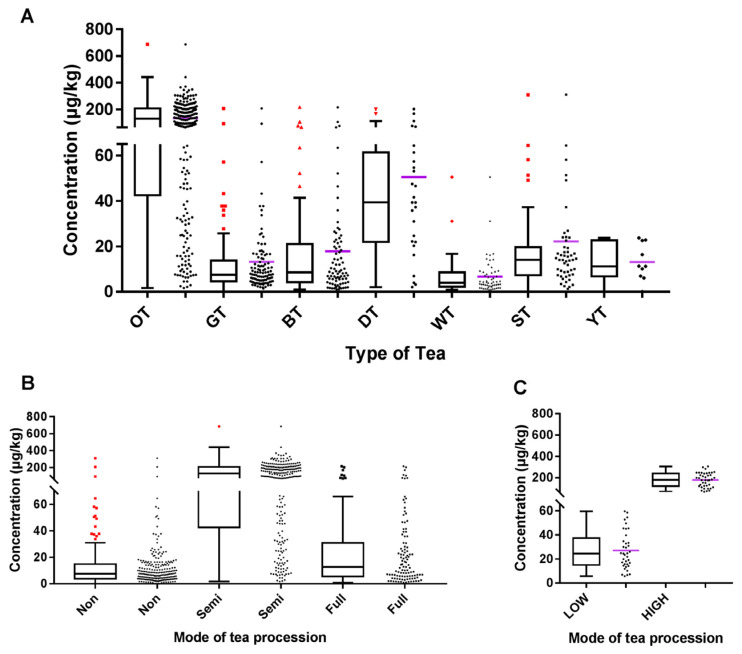
Concentration levels of 4-MEI presented by box and whisker plots: (**A**) among 7 different types of tea; (**B**) among 3 different modes of tea processing; and (**C**) between low and high heat treatment processing.

4-MEI is the final product of the Maillard reaction in tea processing, and its concentration level will be greatly affected by tea processing methods. Herein, a total of 570 tea samples were divided into three main types of tea processing according to the difference in fermentation degrees, including non-fermented tea, e.g., green tea (*n* = 109), white tea (*n* = 56), yellow tea (*n* = 10), scented tea (*n* = 51), semi-fermented tea, e.g., oolong tea (*n* = 237), and fermented tea, e.g., black tea (*n* = 80) and dark tea (*n* = 27). The mean, minimum, maximum, and median concentrations of 4-MEI in all 570 tea samples are presented in Table 2. Among the samples analyzed, 567 samples (99.5%, 567/570) contained 4-MEI, indicating the widespread occurrence of 4-MEI in tea samples. 4-MEI was detected in 100% of the analyzed samples of oolong tea, dark tea, black tea, and white tea. The detection frequency of scented tea, green tea, and yellow tea was 98.0% (50/51), 99.1% (108/109), and 90.0% (9/10), respectively. In previous studies, 4-MEI was also found in all tea samples, although the number of samples was small [17,19,33,40]. In agreement with previous studies carried out in different food categories [17,19,33,40], this study confirmed that 4-MEI was frequently found in tea samples. Among all the tea samples, the highest concentration of 4-MEI was recorded in an oolong tea sample (687 μg/kg). The mean concentrations of 4-MEI (66.6 μg/kg) in all of the samples analyzed in this study were higher than that of tea samples in Belgium (14.0 μg/kg) and Turkey (37.9 μg/kg). Meanwhile, the mean concentrations of 4-MEI in seven different types of tea were observed, as shown in Figure 2A, with the order of oolong tea (136 μg/kg) > dark tea (46.7 μg/kg) > scented tea (22.2 μg/kg) > black tea (17.9 μg/kg) > green tea (13.2 μg/kg) ≈ yellow tea (13.1 μg/kg) > white tea (6.7 μg/kg). According to the Kruskal–Wallis H test, a significant difference in the concentration levels of 4-MEI was observed in the different tea categories (*p* < 0.05, Kruskal–Wallis statistic = 297.2). Dunn’s multiple comparisons test was further performed, and the results showed that 4-MEI concentrations of samples obtained from different tea categories could be distinguished into three different fermented processes (Table S1). Additionally, three different fermented processes of tea were used to compare the amount of 4-MEI. The mean 4-MEI concentrations in non-fermented, fully fermented, and semi-fermented tea were 13.6, 26.1, and 136 μg/kg, respectively (Figure 2B). After further statistical analysis, there was a significant difference in the mean concentration of 4-MEI in the three different fermented processes of tea manufacturing (*p* < 0.05, Kruskal–Wallis statistic = 268.5). Furthermore, Dunn’s multiple comparisons test was performed, and the results are shown in Table S2.

Compared with other types of food, the content of 4-MEI in non-fermented tea and fully fermented tea is comparable to that in most beverages [17,18,40]. It is worth noting that the mean 4-MEI concentration in semi-fermented tea samples was higher than that of other processed foods [17] but lower than that of coffee samples [20]. In general, higher process temperatures lead to higher concentrations of 4-MEI in oolong tea. To this end, we are interested in whether the 4-MEI concentration in oolong tea is directly affected by the temperature in the process of tea preparation. It is expected that the processing temperature leads to the formation of 4-MEI via Maillard-driven pathways. Therefore, two batches of oolong tea samples at different processing temperatures, i.e., low-temperature processing (LT) and high-temperature processing (HT), were analyzed. As shown in Figure 2C, the concentration of 4-MEI in the LT batch samples was significantly lower than that in the HT batch samples (Mann–Whitney test, *p* < 0.0001; mean_LT_ ± SD = 27.1 ± 15.5 μg/kg, median_LT_ = 24.5 μg/kg; mean_HT_ ± SD = 179 ± 66.8 μg/kg, median_HT_ = 180 μg/kg), indicating that higher baking temperatures can lead to higher 4-MEI contamination in oolong tea.

### 3.4. Transfer Rate of 4-MEI from Dry Tea into Infusion

**Figure 3:**
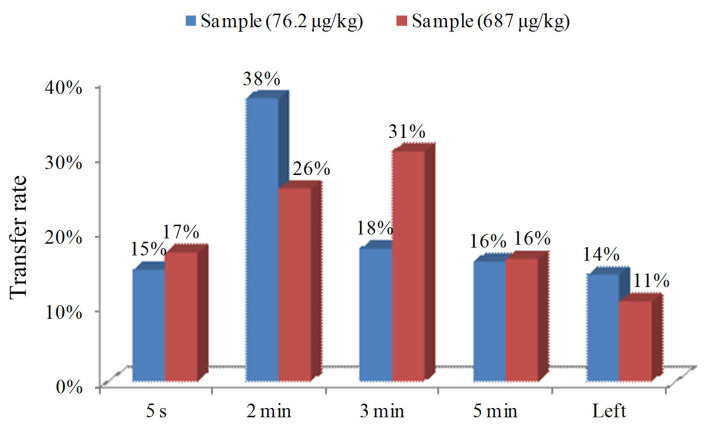
Transfer rate of 4-MEI from dry tea to infusion for two different concentration tea samples.

The results of the transfer rates for 4-MEI from dry tea to infusion with two different concentrations (76.2 and 687 μg/kg) are presented in Figure 3. In the habit of drinking oolong tea, the first short time (5 s) of tea brewing is often used to discard the soaking solution to remove some attachments on the surface of residual tea. The results showed that the first 5 s of brewing helped to remove 4-MEI to some extent, with the high and low concentrations of tea reaching 17% and 15%, respectively. The following three times of brewing can enable the 4-MEI in oolong tea of high and low concentrations to be extracted to 73% and 72%, respectively. However, there were still figures of 11% and 14% for 4-MEI residues in the high and low concentrations of spent tea leaves, respectively. Thus, the sum of the residues of 4-MEI in high and low concentrations of tea infusion was calculated as the total intake of 4-MEI. No obvious difference in transfer rate from dry tea to infusion was observed in this study for different concentration residues in tea, so 72% of the transfer rate was used for the following exposure assessment.

### 3.5. Dietary Exposure of 4-MEI for Chinese Tea Consumers

Table 3 gives an overview of the estimated tea intake exposure of 4-MEI for Chinese tea consumers. As an example for the average intake scenario, the DID of 4-MEI in non-fermented tea was lower (0.04–0.41 μg/day) than that in semi-fermented (0.33–0.98 μg/day) or fully fermented tea (0.18–1.16 μg/day). In three different intake scenarios (the average, the median, and the maximum exposure scenarios), these DID values were low when compared with the limit of 29 μg/day of NSRL set by the OEHHA [28]. However, it cannot be denied that if other sources of 4-MEI are considered, especially those using ammonia caramel as an additive, the total daily dose of 4-MEI in humans is clearly higher. In Folmer et al.’s study [41], the average DID values for the US adult population aged 18 years and older were estimated to be 33, 50, and 93 μg/day from low, average, and high exposure scenarios, respectively. To our knowledge, there are currently no studies evaluating 4-MEI exposure through the daily intake of tea. Therefore, the results of this study may contribute to our understanding of future health risks of exposure to 4-MEI through daily tea-drinking habits.

### 3.6. Strengths and Limitations of This Study

The strengths of this study include the establishment of a simple and feasible analytical method, allowing for the extraction of highly polar 4-MEI from complex tea matrices. This method, based on the traditional LLE technique and relying on the cold-induced phase separation of a acetonitrile–water system, achieves effective purification and enrichment of the strongly polar 4-MEI, thereby providing quality assurance for the detection of 4-MEI in a large number of real tea samples in this study. Although several studies on the analysis of 4-MEI in tea have been reported, the types and quantities of tea samples studied were insufficient. This study fully covered all types of tea samples, expanded the sample size, and fully analyzed the occurrence of 4-MEI among different tea types. Additionally, this study is the first to conduct an exposure assessment of the health risk of 4-MEI among Chinese tea consumers based on the occurrence of 4-MEI in Chinese tea. A limitation of this study is the use of an exposure assessment approach. Although the study used a semi-probabilistic method for exposure assessment, the exposure assessment of 4-MEI was only conducted around a few data distribution points in terms of consumption and contamination levels, so it was not possible to comprehensively obtain specific exposure data for different tea consumption groups. In addition, this study also identified a relationship between high contamination levels of 4-MEI and heat treatment processes. However, it should be noted that it is not yet clear as to which specific heat treatment process at which step is the key factor leading to the formation of 4-MEI, which requires further research. Another limitation of this study is that the relatively small sample sizes of some types of tea may reduce their statistical power to detect significant differences. Despite these limitations, this study contributes to our understanding of the occurrence of 4-MEI in different types of tea, especially the need to pay attention to 4-MEI contamination in oolong tea.

## 4. Conclusions

This study demonstrates a simple, reliable, and highly sensitive analytical method for 4-MEI in tea. This method solves the problems of matrix interference and cumbersome sample preparation faced by previous methods. Using this method to determine the low content of 4-MEI in tea makes this easier, while also protecting the LC-MS used from serious contamination by caffeine and other components. The present study analyzed the 4-MEI concentrations of different types of tea samples from typical tea-producing regions in China. The results showed that a relatively higher contamination concentration was observed in oolong tea, and higher levels of 4-MEI were found during its higher heat treatment process. Further exposure assessment of 4-MEI in tea found that the exposure level and health risk of 4-MEI of Chinese tea consumers are relatively low based on the current study.

## Figures and Tables

**Table 1:** Results of method validation for 4-MEI in tea.

Linear Range (μg/L)	R^2^	LOD (μg/kg)	LOQ (μg/kg)	Spiked Level (μg/kg)	Average Recovery (%, *n* = 6)	RSD (%)	Matrix Effect (*n* = 3)
0.1–100	0.9994	0.3	0.8	1.0	92.5	8.6	0.91 ± 0.04
				10	109	7.2	
				100	106	4.3	

**Table 2:** Statistical analysis of detected 4-MEI concentrations (μg/kg) from the different tea categories.

Group	Categories	Detection Frequency (%)	Mean ± SD	Median (Min–Max)
Non-fermented tea (*n* = 226)		98.7	13.6 ± 26.6	7.7 (ND–310)
	Green tea (*n* = 109)	99.1	13.2 ± 22.5	7.5 (ND–207)
	White tea (*n* = 56)	100	6.7 ± 8.1	4.0 (0.8–50.5)
	Yellow tea (*n* = 10)	90.0	13.1 ± 8.1	11.3 (ND–23.7)
	Scented tea (*n* = 51)	98.0	22.2 ± 43.4	14.1 (ND–310)
Fully fermented tea (*n* = 107)		100	26.1 ± 36.9	12.8 (1.0–216)
	Black tea (*n* = 80)	100	17.9 ± 29.0	8.6 (1.0–216)
	Dark tea (*n* = 27)	100	46.7 ± 9.0	39.3 (2.1–203)
Semi-fermented tea (*n* = 237)		100	136 ± 104	130 (1.7–687)
	Oolong tea (*n* = 237)	100	136 ± 104	130 (1.7–687)
Total	*n* = 570	99.5	66.6 ± 91.8	ND–687

Note: *n*, number of samples; SD, standard deviation; min, minimum; max, maximum; ND, non-detected value.

**Table 3:** 4-MEI exposure levels (μg/day) from the three different tea consumer population groups using a semi-probabilistic approach.

Population Group	Average Scenario						Median Scenario						Maximum Scenario					
	Avg.	P50	P90	P95	P97.5	Max	Avg.	P50	P90	P95	P97.5	Max	Avg.	P50	P90	P95	P97.5	Max
Non-fermented tea	0.04	0.02	0.11	0.16	0.24	0.41	0.02	0.01	0.06	0.09	0.14	0.23	0.87	0.38	2.48	3.73	5.58	9.31
Semi-fermented tea	0.43	0.33	0.97	0.98	0.98	0.98	0.41	0.32	0.94	0.94	0.94	0.94	2.18	1.68	4.93	4.94	4.94	4.94
Fully fermented tea	0.18	0.19	0.37	0.47	0.92	1.16	0.09	0.09	0.18	0.23	0.45	0.57	1.53	1.56	3.08	3.89	7.59	9.63

Note: Avg., average; max, maximum.

## Data Availability

The data used to support the findings of this study can be made available by the corresponding author upon request.

## Supplementary Materials

The following supporting information can be downloaded at: https://www.mdpi.com/article/10.3390/toxics11110916/s1. Table S1: Dunn’s multiple comparisons test for the concentrations of 4-MEI among seven types of tea in China; Table S2: Dunn’s multiple comparisons test for the concentrations of 4-MEI among three different modes of tea processing in China; Figure S1: Maillard reaction in the baking process of tea.
