# Supplementary material for: The Application of Cold-Induced Liquid–Liquid Extraction for the Determination of 4-Methylimidazole in Tea and Associated Risk Assessment for Chinese Tea Consumers
Source: Toxics. 2023 Nov 9;11(11):916. doi: 10.3390/toxics11110916 (PMC10674203; doi:10.3390/toxics11110916)
Supplement: Supplementary file 1 [file toxics-11-00916-s001.zip › toxics-2695091-supplementary.pdf]

Supplementary Materials: The Application of Cold-Induced Liquid–Liquid Extraction for the Determination of 4-Methylimidazole in Tea and Associated Risk Assessment for Chinese Tea Consumers

Shaohua Li, Lian Wang, Dawei Chen and Hong Li

Table S1. Table S1: Dunn's multiple comparisons test for the concentrations of 4-MEI among seven types of tea in China.

Dunn's multiple comparisons test	Mean rank diff.	Significant?	Summary	Adjusted P Value
OT vs. GT	240.5	Yes	****	<0.0001
OT vs. BT	223.4	Yes	****	<0.0001
OT vs. DT	89.24	No	ns	0.1603
OT vs. WT	307.7	Yes	****	<0.0001
OT vs. ST	186.1	Yes	****	<0.0001
OT vs. YT	204.5	Yes	**	0.0025
GT vs. BT	-17.06	No	ns	>0.9999
GT vs. DT	-151.2	Yes	***	0.0004
GT vs. WT	67.22	No	ns	0.2740
GT vs. ST	-54.36	No	ns	>0.9999
GT vs. YT	-35.99	No	ns	>0.9999
BT vs. DT	-134.2	Yes	**	0.0053
BT vs. WT	84.28	No	ns	0.0696
BT vs. ST	-37.3	No	ns	>0.9999
BT vs. YT	-18.93	No	ns	>0.9999
DT vs. WT	218.4	Yes	****	<0.0001
DT vs. ST	96.87	No	ns	0.2825
DT vs. YT	115.2	No	ns	>0.9999
WT vs. ST	-121.6	Yes	**	0.0029
WT vs. YT	-103.2	No	ns	>0.9999
ST vs. YT	18.37	No	ns	>0.9999

Note: green tea (GT), oolong tea (OT), black tea (BT), white tea (WT), dark tea (DT), yellow tea (YT), and scented tea (ST).

Table S2. Dunn's multiple comparisons test for the concentrations of 4-MEI among three different modes of tea processing in China.

Dunn's multiple comparisons test	Mean rank diff.	Significant?	Summary	Adjusted P Value
Non vs. Semi	-243.3	Yes	****	<0.0001
Non vs. Full	-53.71	Yes	*	0.0163
Semi vs. Full	189.6	Yes	****	<0.0001

Note: Non-fermented tea (Non), Full fermented tea (Full), Semi-fermented tea (Semi).

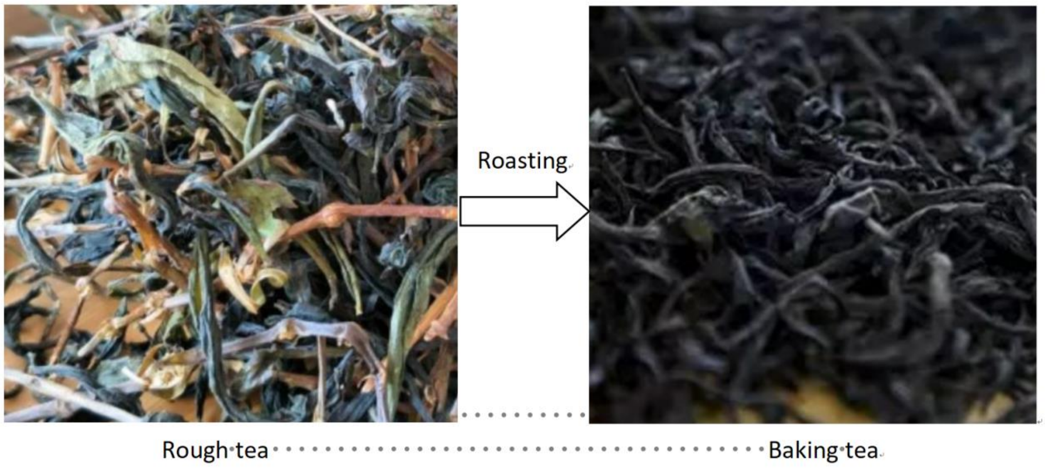

Figure S1. Maillard reaction in the baking process of tea.
